# Mindfulness and Psychological Distress in Kindergarten Teachers: The Mediating Role of Emotional Intelligence

**DOI:** 10.3390/ijerph17218212

**Published:** 2020-11-06

**Authors:** Xiulan Cheng, Ying Ma, Jiaqi Li, Yonghui Cai, Ling Li, Jiao Zhang

**Affiliations:** School of Education, Shaanxi Normal University, Xi’an 710062, China; chengxiulan@snnu.edu.cn (X.C.); cyh@snnu.edu.cn (Y.C.); liling@snnu.edu.cn (L.L.); 845616287@snnu.edu.cn (J.Z.)

**Keywords:** kindergarten teacher, mindfulness, emotional intelligence (EI), psychological distress

## Abstract

Kindergarten teachers are often exposed to great stress. Considering that, mindfulness has been demonstrated to act as a critical role in the psychological well-being of kindergarten teachers. The present study assessed mindfulness in teaching (MT), psychological distress and emotional intelligence (EI) among 511 kindergarten teachers in mainland China and investigated the mediating role of EI to explore the association mechanism between kindergarten teachers’ MT and psychological distress. The major results suggested that kindergarten teachers’ MT was negatively related to their psychological distress (depression, anxiety, and stress). Results of path analyses indicated that the total score of EI and dimension of regulation of emotion (ROE) could serve as significant mediators. The findings suggest that mindfulness might be beneficial to relieve kindergarten teachers’ psychological distress through the mediating role of EI.

## 1. Introduction

Teaching has always been considered as a demanding, challenging and stressful profession [1]. Prior research has supported that teachers’ psychological symptoms, including job-related stress, anxiety, depression and emotional exhaustion were significantly associated with job satisfaction [2], teacher-child relationship [3], child-care quality [4], children’s behavioral problems and higher turnover [5]. The educational objects faced by kindergarten teachers are usually immature and uncontrollable groups, which invisibly increases the difficulty of teachers’ work [6]. Moreover, with the development of the economy, more attention is paid to the quality of individual early education. Thus, kindergarten teachers are now required to complete high-intensity tasks in their daily lives and endure high pressure from society. That includes projecting a myriad of new forms of teaching, participating in practical research, managing disruptive classroom behaviors and promoting children’s social and emotional development [1]. In short, compared with other groups of educators, kindergarten teachers are more vulnerable to psychological distress [1]. It is worth noting that psychological distress is often caused by emotional, work, family and general problems, which leads to weak to severe psychological problems. Considering this scenario, Lovibond and Lovibond proposed the concept of psychological distress, including three dimensions of depression, anxiety and stress [7]. In the present study, we mainly used the concept and measure of psychological distress developed by Lovibond and Lovibond to present the general state of kindergarten teachers’ mental health [7].

Mindfulness, which is significantly associated with a series of mental health outcomes, may be a protective factor of kindergarten teachers’ psychological distress [8]. The concept of mindfulness, rooted in Buddhism, directly refers to an awareness of internal, receptive attention and external experiences at the time of the occurrence [8,9]. Individuals with a high level of mindfulness tend to observe and accept their thoughts and emotions rather than avoiding, suppressing or over-engaging with them [10]. In previous studies, the terms of mindfulness have been described at both trait and state levels. It is categorized as a trait or a disposition with individuals, which tends to be relatively stable between people in the inherent capacity for engagement of mindfulness processes [8,11]. State mindfulness is more focused on the within personal fluctuations in the engagement with the process that rely on the present moment. In this case, it usually occurs naturally in daily life or during mindfulness practice [11]. Many previous studies also demonstrated that mindfulness-based programs could effectively improve individuals’ state mindfulness [12]. Moreover, there is growing empirical evidence supporting the inverse relationship between mindfulness and mental health problems, involving depression, anxiety, stress and emotional symptoms [13], whereas people with higher dispositional mindfulness are hardly affected by habitual patterns [9] and they are more prone to taking opportunities to become aware of, understand and accept negative emotions in current experiences [14].

In recent years, some researchers have begun to pay attention to the association between mindfulness in teaching (MT) and teachers’ mental health [15]. Existing studies suggested that MT is positively related to mental health. Elreda et al. [16] suggested that MT usually promotes teachers’ job satisfaction, ambition and relieves teachers’ perceived stress and improves their ability to be emotionally supportive. Mindful educators tend to have better sleep quality and experience less stress [17]. Researchers also found that teachers having a higher level of dispositional mindfulness was related to better quality teacher-student relationships and lower depressive symptoms, which acted as a partial mediator between greater mindfulness and lower conflict among teachers [18]. Going further, mindfulness could have impacts on teachers’ emotional support in class and relationships with children and play, which have a big impact on student outcomes [19,20]. In addition, some previous related research suggested that mindfulness-based interventions (MBIs) could help reduce stress through recognizing and regulating emotions [21]. The effective outcomes of MBIs on teachers’ mental health include the improvement of well-being, self-compassion, emotional awareness in the classroom and reduction of stress, burnout and depression [22]. Based on prior research, it can be concluded that mindfulness is significantly related to teachers’ psychological symptoms and that a mindful teacher may perform and feel better at work. Nevertheless, the existing limited studies have focused on the samples of primary and secondary school teachers, and there is a dearth of research on this aspect when it comes to kindergarten teachers.

Considering the mechanism underlying mindfulness and psychological symptoms, some researchers have already put forward certain mediating variables, such as emotional regulation [23], self-esteem [24], rumination [25] and autonomy [26]. Emotional Intelligence (EI) as a potential psychological factor might also help to explain the potential mechanism underlying the association between kindergarten teachers’ mindfulness and psychological distress. Salovery [27] hold that EI should be defined as an ability that includes reflectively regulating, understanding, absorbing, perceiving and expressing emotions. Wong and Law [28] proposed four dimensions of EI: (i) self-emotional appraisal (SEA), which means the capability to observe, realize and state an individual’s profound emotions appropriately and naturally; (ii) others’ emotional appraisal (OEA), which is related to the individual’s capability to be aware of and understand the emotion of other people accurately in the surrounding settings; (iii) use of emotion (UOE), which is the capability to use emotions for facilitating one’s constructive behaviours and personal performance; (iv) regulation of emotion (ROE), which means the capability to control and regulate emotions and may be the determinant to mitigating one’s psychological distress.

Generally speaking, there is a positive association between mindfulness and EI [8]. To some extent, both have similar concerns and highly emphasize individual ability to observe, comprehend and regulate their emotions [29]. Characteristics of mindfulness contain clarity of awareness, non-judgment, orientation to the present, openness and acceptance to positive and negative emotions [9]. Mindful individuals tend to have much better cognitive flexibility and could be more sensitive to the emotions of other people around them, an aspect that might be beneficial to the clarity of emotional state [30]. In addition, individuals with greater mindfulness may accept any thoughts and emotions with self-compassion rather than engaging in ruminative thinking of negative emotions excessively [25]. In this sense, mindfulness is regarded as an association with SEA and OEA. Furthermore, mindfulness emphasizes attention to a present experience and facilitates the choice of behaviors, including one’s needs, interests and values [8]. In addition, mindfulness could help to generate more lucidity and vividness of a current experience and stimulate internal sensation to be intimately in contact with the present life. This would strengthen the ability of self-regulation as a consequence and be accompanied by sustained attention sensitivity to psychological, physical and environmental cues [31]. Studies have demonstrated that mindfulness training is closely connected with the amelioration of attention functions, cognitive flexibility and problem-solving features [32]. That said, individuals would perform well on their current tasks in positive emotions, and the ability of UOE and ROE could even be enhanced [33,34].

EI has also been demonstrated to be related to psychological health across different samples. Individuals with higher EI show more positive mood [35], greater satisfaction with life [36], higher levels of well-being [37] and lower levels of psychological stress [23]. Additionally, Schutte and Malouff [38] found that EI proved to be a partial mediator among subjective well-being with mindfulness in university students and perceived stress in Chinese adults [31]. In the study of Wang and Kong [39], EI also significantly mediated the relationship between mindfulness and perceived distress in a sample of adolescents. However, even with a few studies regarding this aspect, EI is considered as multi-dimensional construct [27], which aspects of EI play a more important role in the relationship between mindfulness and psychological distress is unclear. Therefore, it is necessary to explore the mediating role of EI and its dimensions between mindfulness and psychological distress.

To sum up, this study might help clarify the relationships of MT, EI and psychological distress among kindergarten teachers and investigates the specific mechanism of EI and its dimensions as mediating variables. The following hypotheses are tested: (i) MT is positively associated with EI (including the EI total score and subscale scores), (ii) MT is negatively associated with psychological distress, and (iii) EI (including the EI total score and subscale scores) mediates the effect of MT on psychological distress.

## 2. Materials and Methods

### 2.1. Participants and Procedure

Participants in this study were 511 kindergarten teachers from Shaanxi Province, China, aged from 18 to 57 years old (M = 30.39, SD = 7.46). The average work experience was 9 years (M = 8.83, SD = 7.63), and 490 were females (95.9%). Participants’ education levels were distributed as high school and below (*n* = 131, 25.6%), bachelor’s degree (*n* = 352, 68.9%) and master’s degree and above (*n* = 28, 5.48%). This was similar to the gender ratio, age distribution and educational background compared with kindergarten teachers in other studies [6,40]. This research followed the principles of the Declaration of Helsinki. Before the survey, teachers receivedinformed consent, and the ethics committee approved all the process of the present study.

### 2.2. Measures

#### 2.2.1. Mindfulness

The Mindfulness in Teaching Scale (MTS) [41] is a 14-item scale consisting of two dimensions measuring teacher intrapersonal mindfulness (e.g., When I am teaching, I find myself doing things without paying attention) and teacher interpersonal mindfulness (e.g., I am aware of how my moods affect the way I treat my students). Participants were asked to rate on a 5-point Likert-type scale (1 = almost never to 5 = almost always). The higher total score indicated higher levels of mindfulness in teaching. The Chinese version of the MTS was verified with good validity and reliability [42]. The scale had a Cronbach alpha coefficient of 0.82 in this study, which showed good internal consistency.

#### 2.2.2. Emotional Intelligence

The 16-item Wong Law Emotional Intelligence Scale (WLEIS) was used to measure emotional intelligence in the study [28], which included four dimensions: self emotion appraisals (SEA: “I have a good understanding of my own emotions”); others’ emotion appraisals (OEA: “I have a good understanding of the emotions of people around me”); regulation of emotion (ROE: “I am quite capable of controlling my own emotions”); and use of emotion (UOE: “I would always encourage myself to try my best”). Items were rated on a 5-point Likert-type from 1 (strongly disagree) to 5 (strongly agree). A higher score reflected a higher level of emotional intelligence. The Chinese version of the WLEIS has been validated with good psychometric properties [43]. In this study, the Cronbach alpha coefficients of the four subscales were SEA = 0.84, OEA = 0.80, ROE = 0.84 and UOE = 0.77, and the total scale was 0.92, which indicated good internal consistency.

#### 2.2.3. Depression Anxiety Stress Scale

The Depression Anxiety Stress Scales-21 (DASS-21) is a 21-item measure, including three subscales (depression, anxiety, and stress) [7], and each subscale includes 7 items. Participants rated the frequency they experience symptoms related to depression (DASS-D; e.g. “I couldn’t seem to experience any positive emotion at all” ), anxiety (DASS-A; e.g. “I experienced trembling [e.g. in the hands]”) and stress (DASS-S; e.g. “I found it hard to wind down”) on a 4-point scale, from 0 (‘not for me at all’) to 3 (‘very or most of the time’). The higher the total score, the higher the level of psychological distress. The factor structure of the Chinese version of DASS-21 was supported by previous studies, and the internal consistency of each subscale was above 0.75 [44]. In this study, Cronbach’s alphas of the DASS-21 subscale were 0.86 (depression), 0.77 (anxiety) and 0.80 (stress). The Cronbach alpha coefficient of the total scale was 0.92. Therefore, the scale showed good internal consistency.

### 2.3. Data Analyses

Descriptive data of the main variables were reported (means and standard deviations), and then Pearson’s correlations were performed to test the relationship among variables. The bootstrap method was used in a procedure of 5000 samples to analyze the mediating effects via the SPSS macro ’process’ [45]. In this process, a 95% confidence interval would be generated to test the significance of the indirect effect between MT and psychological distress through mediating roles of EI (including the EI total score and subscale scores). The indirect effects were identified as significant if zero does not exist between the lower and upper confidence intervals (CIs).

## 3. Results

Descriptive statistics and Pearson’s correlations among variables were presented in Table 1, including mean values, standard deviations and correlations. MT was positively related to the total score of EI and the scores of subscales including SEA, OEA, UOE and ROE. Conversely, MT was negatively related to the psychological distress total score and subscales of depression, anxiety and stress. In addition, the total score of EI and its subscales were negatively correlated with psychological distress and its dimensions.

**Figure 1:**
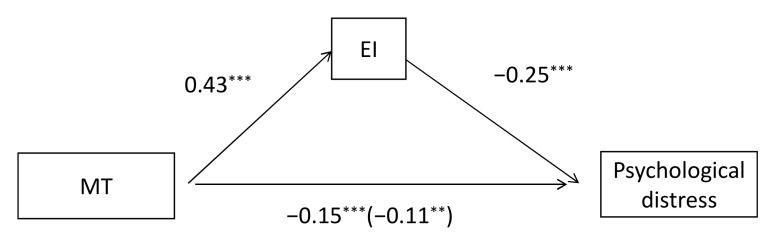
Mediator model examining the indirect relationship between MT and psychological distress through EI total score. Numbers in parentheses represent indirect effects for mediator variable. ** *p* < 0.01.

We first explored the mediating role of EI total score in the relationship between MT and psychological distress. As shown in Figure 1, the results of regression showed that MT had a significant positive effect on EI, and EI had a significant negative effect on psychological distress. The indirect effect of MT on psychological distress was significant when EI total score was the mediating variable (Figure 1 and Table 2). In addition, the direct effect of MT on psychological distress was still significant after accounting for the mediator variable of EI total score (Figure 1). The results indicated that EI total score could serve as a partial mediating variable between MT and psychological distress.

**Figure 2:**
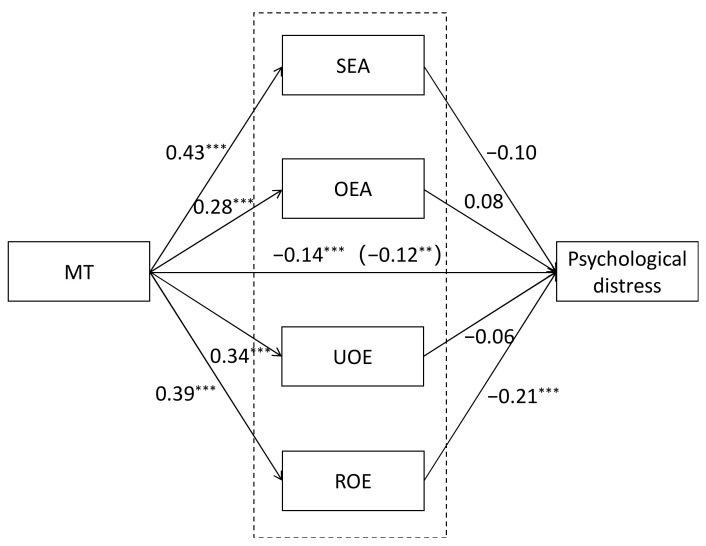
Multiple mediator model examining the direct and indirect relation of MT and psychological distress. Numbers in parentheses represent indirect effects. Note: all regression coefficients are standardized. SEA, ROE, UOE and OEA are the subscales of the Wong Law Emotional Intelligence Scale. ** *p* < 0.01.

We further tested the mediating effects through the four dimensions of EI in a multiple mediation model. As seen in Figure 2, the results of regression showed that MT had significant effects on each mediator variable: SEA, OEA, UOE and ROE. Three of the mediators, SEA, OEA and UOE, had non-significant effects on psychological distress. The other, ROE, had a significant effect on psychological distress. After including the four subscales as mediators in the model, the total indirect effect was still significant, but only the dimension of ROE served as a significant mediating variable compared with other dimensions of EI. What is more, the direct effect of MT on psychological distress was still significant after accounting for mediating variables (Figure 2). These results indicated that EI dimensions, especially ROE could partially mediate the relationship between MT and psychological distress.

## 4. Discussion

This study mainly examined whether MT would be negatively associated with psychological distress and positively associated with emotional intelligence in a sample of kindergarten teachers. In addition, we also investigated the mediating role of emotional intelligence between kindergarten teachers’ MT and psychological distress. The present study might helpe to closely investigate the mediating role of different dimensions of EI between kindergarten teachers’ MT and psychological distress. The results partially supported the hypotheses. The link between MT and psychological distress was mediated by the total score of EI and its subscales of ROE.

The outcomes of this research supported that MT was inversely related to teachers’ psychological distress, which was consistent with previous research that proved a negative association between kindergarten teachers’ mindfulness and negative emotions (e.g., emotional exhaustion and depression) [46]. A previous study suggested that teachers’ mindfulness might help teachers build positive relationships and promote an emotionally supportive classroom climate in the face of stressors, and deal with complicated situations more proactively or more adaptively. Mindful teachers also responded to classroom circumstances and students’ needs with openness, acceptance and compassion [19,41,47]. Therefore, teachers who have higher dispositional mindfulness would be likely to experience low levels of psychological distress.

As expected, the results support that MT was positively related to EI and its subscales, which was consistent with previous studies which reported that mindful attention awareness was positively related to EI [31,39]. Wang and Kong [39] also suggested a significant positive association between mindful attention awareness and the total score of EI. The capability to recognize individual own emotions is a central aspect of EI, also named SEA, which is in line with a person’s mindfulness and especially related to the self-acceptance of mental state [8]. Therefore, compared with other dimensions, the positive correlation between mindfulness and SEA seemed the most significant, which was consistent with previous studies [31]. People with a higher degree of dispositional mindfulness are inclined to pay attention to their internal experience, external judgments and keep awareness of every moment [8,48]. Our results also showed that MT was also positively related to OEA. An existing study regarding this indicated that mindfulness helped individuals to perceive their own emotions as well as others’ emotions more accurately and effectively [31,38]. In addition, the present findings suggested that MT could positively predict UOE and ROE. Recent studies have demonstrated that mindfulness training was closely connected with the amelioration of attention functions, cognitive flexibility and problem-solving features [49]. In this case, mindful individuals are more likely to motivate themselves to regulate their emotions with the aim to achieve better performance. Some other researchers also found that mindfulness skills were associated with perceived self-control of negative emotions, which promoted a sense of control, and greater perceived control was associated with greater self-efficacy and work performance [32]. Furthermore, mindfulness focuses on consciousness and open awareness to the current experience, nurturing a stronger sense of self-control and behavioural regulation [29]. Kindergarten teachers usually need a high degree of emotional control and use due to the particularities involved in their profession [1]. Wang and Kong [39] also found that mindfulness was positively related to UOE and ROE. Thus, MT might positively assist teachers in promoting a higher level of UOE and ROE, which is consistent with previous studies [31].

The present results also showed that the EI total score played a mediating role between MT and psychological distress. This finding was consistent with those of Wang and Kong [39], who found that the total score of EI could mediate the relationship between mindfulness and psychological distress. We further extended the mediation model to the MT and psychological distress relationship in kindergaten teachers. The results of multiple mediation analyses suggested that only the dimension of ROE could play a significant mediating role between MT and psychological distress compared with other dimensions, which was not in line with our hypothesis. That is to say, the self-regulated functioning inherent in mindfulness relates to the ROE component of EI. This was consistent with previous studies about behavioural and neuroimaging evidence whereby emotion regulation has been linked with mindfulness [50]. In the study of Bao and Kong [31], dimensions of UOE and ROE could also mediate the relationship between mindfulness and perceived stress. Our result suggested that especially ROE might be an essential mechanism by which MT might help reduce psychological distress. Judging from the essential characteristics of ROE, it emphasizes the capability to control and regulate emotions, and it is the determinant to mitigating one’s psychological distress [28], which is crucial for one’s emotional experiences and adaptation to socioemotional challenges [51,52]. What is more, ROE was related to awareness, adapt, clarity and acceptance of emotions, as well as the ability to control emotional impulse and act in desired ways [53]. In other words, people with a higher degree of emotional regulation have a clearer cognition of their own emotions, find it easier to recover from negative situations and adjust their emotions flexibly, thereby avoiding emotional dysregulation to the greatest extent. As a result, ROE could effectively help kindergarten teachers cope with occupational stress with positive emotions, which proved the significant mediating role of ROE between EI and psychological distress.

We proposed the possible reasons for these results here. Judging from the essential characteristics of OEA, the ability of OEA emphasizes individuals’ ability to perceive and comprehend emotions of other people and thereby to predict the possible emotional expression and emotional response of others around them [54], paying more attention to external people or situations, and so contributes relatively little in the process of mindfulness alleviating one’s own psychological distress. As for SEA, which means the capability to recognize personal and individual emotions, it is in line with a person’s mindfulness and especially related to the self-awareness of mental state. Both SEA and OEA emphasize the appraisal of psychological quality. However, relieving psychological distress is often achieved through the direct regulation of psychological mechanisms. Compared with ROE, SEA [31] and OEA had a smaller association with psychological distress. UOEwas closely connected with the amelioration of attention functions, cognitive flexibility, self-efficacy and problem-solving features [32,49], which helped to enhance their performance and then indirectly relieved ones’ psychological distress. Thus, ROA had a smaller association with psychological distress. Moreover, considering that OEA, SEA and UOE refer to the appraisal and use of emotions, and given that such dimensions are negatively correlated with psychological distress, it is possible that such dimensions can have a direct effect on reducing psychological distress by allowing people to have better social interactions and increase their performance, but not through more self-awareness and a regulatory process as mindfulness implies. Further studies should shed some light on this interesting research avenue and explore the effect of EI dimensions on different outcomes or dependent variables.

This study still has some limitations worth noting. First, the methods of this study were correlational and cross-sectional. Although previous studies have verified the impact of mindfulness on psychological distress, future studies could use longitudinal data to conduct cross-lag analysis to eliminate reverse causality to enhance the reliability of the results. Thus, longitudinal design or interventions are suggested in the future to provide more precise information about the direction of the causal relationship between kindergarten teachers’ MT and emotional intelligence and psychological distress. Second, the data were dependent on self-report measures. Despite the excellent reliability and effectiveness of the tools used here, self-reported measures are inherently subjective and may cause bias (e.g., social expectations). A variety of assessment methods could be used to reduce the influence of subjectivity, such as behavioural measurements and so on. Third, the mediation effect of the study was relatively small. This may be due to the existence of various mediating mechanisms between MT and psychological distress. Some other mediating variables may help explain this relationship, such as attachment style [33], rumination [55] and psychological capital [56]. We mainly explored the potential relationship between MT and psychological distress of kindergarten teachers from the perspective of EI. Therefore, it is important to conduct more research to deepen our understanding of the connection between MT and psychological distress from different theories.

Despite these limitations, the present research provided insight into the internal paths between mindfulness and psychological symptoms in a sample of kindergarten teachers. The present study proposed that kindergarten teachers’ mindfulness exerted effects on psychological distress through emotional intelligence, especially via dimensions of emotional regulation. This result may provided some valuable guidance for kindergarten teachers to implement mindfulness practice. What is more, COVID-19 poses a huge challenge to the work and life of an individual [57,58]. A recent study suggested that mindfulness-based training can effectively mitigate the negative psychological consequences of the COVID-19 outbreak, helping to restore well-being in the most vulnerable individuals [59]. Like other professions [58], kindergarten teachers have also experienced tremendous occupational pressure following the COVID-19 outbreak. Mindfulness skills may enable kindergarten teachers to regulate, control and use their own emotions better and better perceive the emotions of others, thereby enhancing their emotional adaptability and regulating ability [57,58]. Thus, it is necessary for kindergartens to pay attention to the mental state of kindergarten teachers and incorporate mindfulness into the teacher training system, which could help teachers realize the remission of psychological distress and better focus on their work. All in all, the findings are of great significance to the psychological rehabilitation and guidance of kindergarten teachers in the post-COVID era.

## 5. Conclusions

This research investigated the mediating path between kindergarten teachers’ mindfulness, emotional intelligence and psychological distress and found that: (i) MT was positively associated with EI (including the EI total score and subscale scores), (ii) MT was negatively associated with psychological distress, and (iii) EI and its dimension, especially ROE, mediated the effect of MT on psychological distress. These results provide some valuable guidance for kindergarten teachers to implement mindfulness, which helps achieve psychological health. In the field of early childhood education, mindfulness intervention as a preventive therapy may help kindergarten teachers effectively recognize and regulate emotions, thereby reducing their psychological distress. All in all, this is of great significance for promoting the professional mental health of kindergarten teachers. Additionally, the present findings may provide a better understanding of new issues and challenges for occupational health psychology in the post-COVID era.

## Figures and Tables

**Table 1:** The means, standard deviations and inter-correlations of all the studied variables.

Variables	Means (SD)	1	2	3	4	5	6	7	8	9	10
1.MT	3.86 (0.59)	1									
2.EI	4.08 (0.55)	0.43 **	1								
3.SEA	4.31 (0.62)	0.43 **	0.84 **	1							
4.OEA	3.97 (0.68)	0.28 **	0.80 **	0.57 **	1						
5.UOE	4.08 (0.64)	0.34 **	0.85 **	0.62 **	0.53 **	1					
6.ROE	3.98 (0.68)	0.39 **	0.87 **	0.65 **	0.55 **	0.70 **	1				
7.Psychological Distress	0.65 (0.46)	−0.26 **	−0.31 **	−0.29 **	−0.16 **	−0.28 **	−0.33 **	1			
8. Depression	0.51 (0.48)	−0.31 **	−0.34 **	−0.31 **	−0.21 **	−0.30 **	−0.33 **	0.92 **	1		
9. Anxiety	0.63 (0.48)	−0.21 **	−0.24 **	−0.23 **	−0.12 **	−0.21 **	−0.25 **	0.92 **	0.78 **	1	
10.Stress	0.80 (0.52)	−0.21 **	−0.28 **	−0.24 **	−0.13 **	−0.25 **	−0.32 **	0.93 **	0.77 **	0.78 **	1

Note: N = 511. ** *p* < 0.01. MTS = Mindfulness in Teaching Scale; EI = emotional intelligence, SEA = self emotion appraisals; OEA = others’ emotion appraisals; UOE = use of emotion; ROE = regulation of emotion.

**Table 2:** Indirect effects of MT on psychological distress through mediating variables.

	DASS		
Variables	b	95% CI	*p*
**EI**	−0.11	−0.15, −0.06	<0.01
**SEA**	−0.04	−0.1, 0.01	0.11
**OEA**	0.02	−0.01, 0.06	0.13
**UOE**	−0.02	−0.07, 0.02	0.31
**ROE**	−0.08	−0.14, −0.02	<0.01

Note: N = 511. EI = emotional iIntelligence, SEA = self emotion appraisals; OEA = others’ emotion appraisals; UOE = use of emotion; ROE = regulation of emotion.
